# Effects of Tail Clipping on Larval Performance and Tail Regeneration Rates in the Near Eastern Fire Salamander, *Salamandra infraimmaculata*

**DOI:** 10.1371/journal.pone.0128077

**Published:** 2015-06-11

**Authors:** Ori Segev, Antonina Polevikove, Lior Blank, Daniel Goedbloed, Eliane Küpfer, Anna Gershberg, Avi Koplovich, Leon Blaustein

**Affiliations:** 1 Institute of Evolution and Department of Evolutionary and Environmental Biology, Faculty of Natural Sciences, University of Haifa, Haifa, 3498838, Israel; 2 Department of Plant Pathology and Weed Research, ARO, The Volcani Center, Bet Dagan, 50250, Israel; 3 Technical University of Braunschweig, Zoological Institute, Braunschweig, 38106, Germany; Laboratoire Arago, FRANCE

## Abstract

Tail-tip clipping is a common technique for collecting tissue samples from amphibian larvae and adults. Surprisingly, studies of this invasive sampling procedure or of natural tail clipping – i.e., bites inflicted by predators including conspecifics - on the performance and fitness of aquatic larval stages of urodeles are scarce. We conducted two studies in which we assessed the effects of posterior tail clipping (~30 percent of tail) on Near Eastern fire salamander (*Salamandra infraimmaculata*) larvae. In a laboratory study, we checked regeneration rates of posterior tail-tip clipping at different ages. Regeneration rates were hump-shaped, peaking at the age of ~30 days and then decreasing. This variation in tail regeneration rates suggests tradeoffs in resource allocation between regeneration and somatic growth during early and advanced development. In an outdoor artificial pond experiment, under constant larval densities, we assessed how tail clipping of newborn larvae affects survival to, time to, and size at metamorphosis. Repeated measures ANOVA on mean larval survival per pond revealed no effect of tail clipping. Tail clipping had correspondingly no effect on larval growth and development expressed in size (mass and snout-vent length) at, and time to, metamorphosis. We conclude that despite the given variation in tail regeneration rates throughout larval ontogeny, clipping of 30% percent of the posterior tail area seems to have no adverse effects on larval fitness and survival. We suggest that future use of this imperative tool for the study of amphibian should take into account larval developmental stage during the time of application and not just the relative size of the clipped tail sample.

## Introduction

Amphibian urodeles possess the ability to regenerate body parts including tail, limbs, external gills, jaws, and parts of the eye into complete and functionally restored structures[1]. Lost body parts are often incurred by predation, including by cannibalistic conspecifics (e.g., [2,3,4]). Researchers may also clip amphibian body parts for mark-recapture studies [5,6], stable isotope analysis [7,8],or for obtaining tissue sample for genetic studies. Invasive and non-invasive techniques for sampling amphibian DNA include: tail clipping [9], toe clipping [10], blood puncture [11], skin swabbing [12], and buccal swabbing [13]. The applicability and efficiency of these methods vary among species but most are not suitable for sampling DNA from early development larval stages due to their small size and handling sensitivity. Validating the effect of tail clipping for obtaining tissue samples from amphibian larval stages is particularly important, as these are often the stages that are spatially concentrated and readily available in the field in large quantities unlike the post-metamorphic terrestrial stages.

During the time frame between losing a body part and its complete regeneration, animals may suffer various costs related to reduced performance. In general, there is a positive relationship between the larval tail area and propulsive performance in amphibians [14,15,16]. Losing parts of the tail may impair locomotor performance and hence the ability to escape predators and/or capture prey. Studies comparing the swimming performance of amphibian aquatic stages before and after autotomy suggest that losing approximately 30% of the tail area represents a threshold beyond which a decrease in burst speed occurs [17,18,19]. Reduction in the tail surface area may not only impair larval locomotion but can also reduce the tail attractiveness as a lure diverting the attacks of visual predators' away from the more vulnerable head [20,21,22].

A number of studies manipulated the tail dimensions of anuran tadpoles and monitored locomotor performance or life history traits [18, 21,23,24,25,26]. Few studies exist that assess the fitness effect of a truncated tail in urodele aquatic larval stages [27,28,29] and patterns seen in anurans may not necessarily be extrapolated to urodeles for several reasons. The kinematic patterns of swimming differ between urodele larvae and anuran tadpole [16,30]. Consequently damage to the tail tissues may affect locomotor abilities differently between the two orders. Moreover, the difference in diet requirements and foraging behavior between mostly carnivorous urodele larvae and herbivorous anuran tadpoles may reflect differently on performance and fitness following tail injury. Lastly, modes of tail regeneration appear to differ between urodele larvae and anuran tadpoles [31, 32] and even within urodele species [33].

The aquatic pre-metamorphic larval stages of many complex life cycle organisms including amphibians are sensitive in terms of per capita survival. A major cause for salamander larval mortality in temporary ponds that is characterized by limited food resources and finite water volume is cannibalism both within and between cohorts [34,35,36,37].

*S*. *infraimmaculata* displays a larviparous reproduction mode, giving birth to large broods of larvae with well-developed appendages and tail fins. Larvae use tail movements to generate thrust during swimming. Different levels of damage to the tail tissue have been shown in anuran tadpoles to cause a reduction in swimming performance that in turn causes increased mortality due to cannibalism [38]. Non-lethal tail injuries may also impair fitness-related traits such as size at, and time to metamorphosis that in turn can be carried over to the adult stages and affect, for example, age and size at first reproduction [39,40], or fecundity in the form of egg size and number [41].

We assessed regeneration rates of clipped tail fins in *S*. *infraimmaculata* larvae in the laboratory as a function of age when isolated from potential predation and fed *ad libitum*. We additionally tested the hypotheses that: 1) tail clipping will have a deleterious effect on larval survival particularly shortly after the tail was truncated and had insufficient time to fully regenerate; 2) tail clipping will cause a reduction in mass at, and size at, metamorphosis and will extend the time to metamorphosis due to growth and developmental costs associated with the damaged tail.

## Methods

### Laboratory experiment

We conducted a laboratory study to determine the how tail-fin regeneration rate was related to salamander larval age. Under constant controlled conditions of 21°C, 5 L water, and 12/12 light/dark cycle, we raised 16 ~one-week old salamander larvae to metamorphosis. Larvae were reared indoors and kept individually in plastic containers (Length x Width x Height: 32 x 18 x 11cm) filled with 5 L of aged tap water. Larvae were obtained from two breeding sites in northern Israel: Tel-Dan (33°14'59''N;35°40'62''E), and Manof (32°50'58''N; 35°13'52''E). We used 8 larvae which were deposited in the laboratory by a single gravid female from the Tel Dan population on 5 February 2014, and 8 larvae collected on 9 February 2014 from the temporary pond in Manof. Because the Manof pond was dry before being inundated by rains on 2 February, the maximum age of the larvae when collected was 7 days. Larvae were fed every second day with *Chironomus sp*. larvae—5, 10, and 15 per salamander larva during the first, second, and third month, respectively.

**Fig 1:**
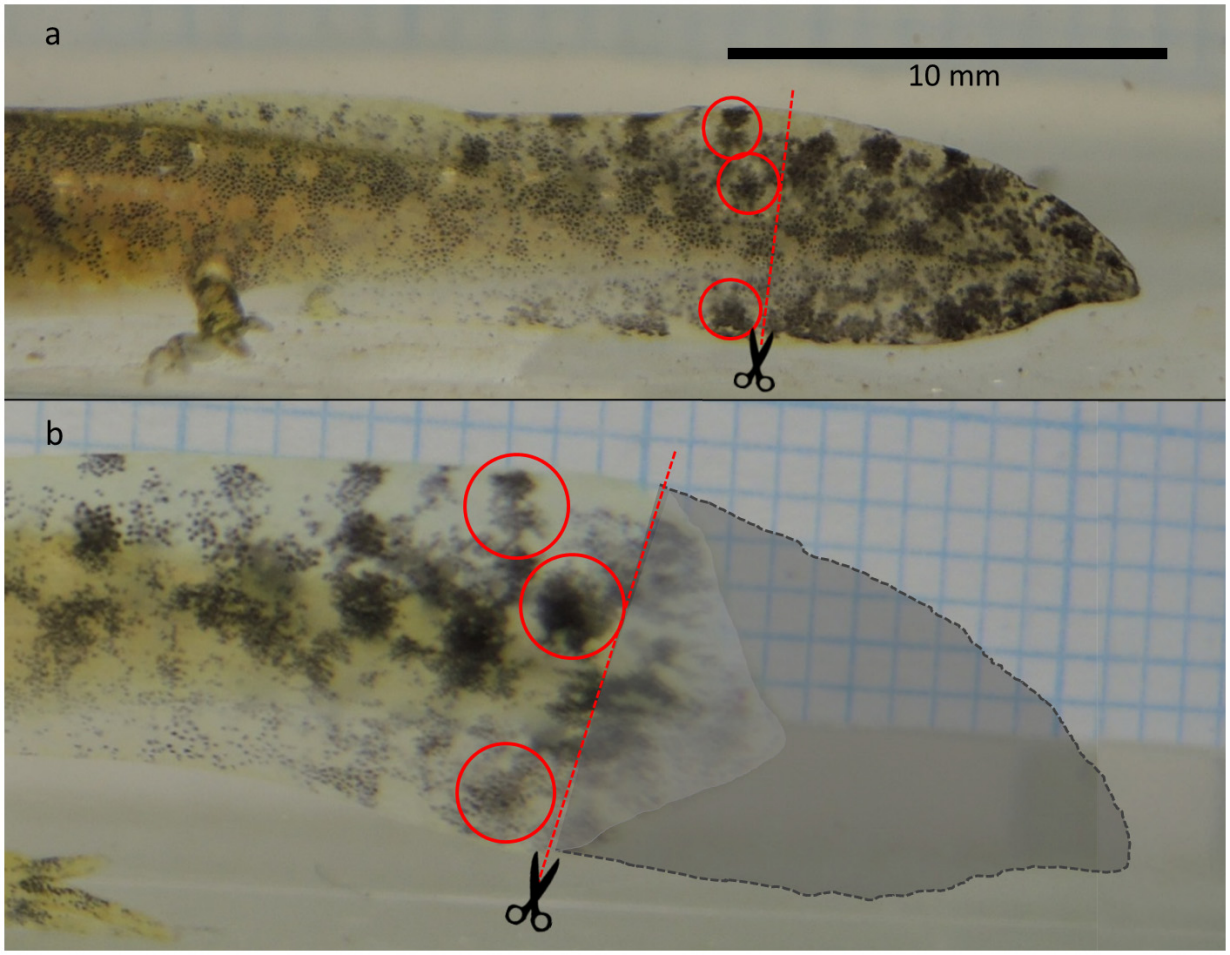
Lateral view of atail of *S*. *infraimmaculata* larva before clipping (a), and following a 37 day interval during which 29.4% of the tail area had regenerated (b). The shaded grey area (Fig 1b) denotes the relative clipped area taking into consideration the increase in total tail area. The position of the vertical incision, denoted by a dashed red line, was determined in Fig 1a relative to the position of the tail spots; a sample of three is enclosed in red circles.

**Fig 2:**
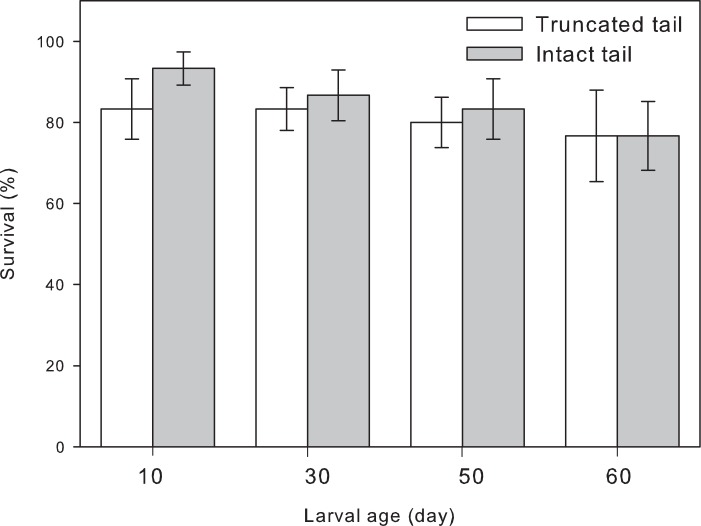
Tail regeneration rate per day of *Salamandra infraimmaculata* larvae versus larval age (day) at the time of tail clipping (N = 16). A polynomial 2^nd^ degree curve explained 48.3% of the variation in the data (F_2, 13_ = 6.07, p = 0.014).

We clipped tail tips of different individuals beginning on 11 February2014 and ending on 1 April 2014. We clipped on average, 28.19% (SE = 3.8)from the posterior tailfin-tip of the16 individual larvae at specific ages counted from date of deposition in days as follows:7 days(4 individuals); 14 days (3 individuals); 21 days(2 individuals); 30 days (2 individuals); 37 days (3 individuals); 44 days (2 individuals). Larvae were photographed laterally using a tripod-mounted camera (Nikon Coolpix P770) in a glass chamber containing clear water and 1mm grid paper that was later used as a scale reference. Each larva was photographed twice-right before tail clipping and after a predetermined time interval to allow regeneration. Regeneration interval had some variation between age groups (Mean = 28.25; SE = 2.5 days). To estimate tail regeneration rate, we used variables 1–4 listed below. We extrapolated the clipped area at *t*_+1_ (5), then divided the ratio between the regenerated area and the clipped area at time *t*_+1_ (6) by the duration of the regeneration interval (Fig 1). Regeneration parameters were estimated blindly by two persons using the software ImageJ [42]. We found a strong, positive monotonic correlation between the estimated regeneration rates of the two independent observers (r = 0.815, p <0.001). The average scores per age group were used for the analysis of age dependent tail regeneration (Fig 2).

$$\text{Total}\;\text{area}\;\text{at}\;\text{time}\;t\text{:}\;X_{t}$$ (1)

$$\text{Clipped}\;\text{tail}\;\text{at}\;\text{time}\;t\text{:}\;Y_{t}$$ (2)

$$\text{Remaining}\;\text{tail}\;\text{area}\;\text{after}\;\text{clipping}\;\text{at}\;\text{time}\;t\text{:}\;Z_{t}=X_{t}-Y_{t}$$ (3)

$$\text{Regenerated}\;\text{area}\;\text{at}\;\text{time}\;t+1\text{:}\;R_{t+1}$$ (4)

$$\text{Clipped}\;\text{area}\;\text{at}\;\text{time}\;t+1\text{:}\;Y_{t+1}=(Y_{t}*Z_{t+1})Z_{t}$$ (5)

$$\text{Ratio}\;\text{between}\;\text{the}\;\text{regenerated}\;\text{area}\;\text{and}\;\text{the}\;\text{clipped}\;\text{area}\;\text{at}\;\text{time}\;t+1:\;R_{t+1}/Y_{t+1}$$ (6)

Polynomial curve fitting was performed using the statistical software JMP version 5.0.1 (SAS Institute Inc., Cary, NC, 1989–2007).

### Outdoor mesocosm experiment

We conducted an outdoor-mesocosm experiment at the University of Haifa campus to assess how posterior tail-fin clipping of *S*. *infraimmaculata* larvae affected larval performance. Ten mesocosms—plastic tubs (Length x Width x Height: 54 x 42 x 21 cm) filled with a combination of aged tap water and rain water—were used in this experiment. In each tub, five approximately equal-sized rocks (total volume displaced: 2.5 L) provided a refuge for the larvae. Water volume (40 L) was kept constant throughout the experiment. The mesocosms were placed under an 85% net shade suspended at the height of 2 m. The tops of the tubs were open to allow insect colonization and other allochthonous input. In addition to colonizing insects, salamander larvae were fed once per week with *Chironomus* larvae (mean wet mass per week: 0.472 g; SE = 0.046 g) per tub.

The larvae used for the experiment were deposited in the laboratory by five gravid females collected from two ephemeral breeding sites (Secher Pool [32°44'04''N; 35°01'52''E] and Manof [32°50'58''N; 35°13'52''E]), and three permanent breeding sites (Ein Alon [32°43'29''N; 35°01'31''E], Ein el Balad [32°43'13''N; 35°04'17''E], and Kaukab Springs [32°49'23''N; 35°14'51''E]) in northern Israel. A single female was collected from each site, and each female gave birth in the laboratory.

On 10 February 2014, 12 larvae from each female were equally and randomly divided into two tubs; each of the 10 tubs contained six sibling larvae—three with a clipped tail and three with an intact tail (overall, 60 larvae). The density used (1 larva per 6.7 L) is well within the density range of *S*. *infraimmaculata* larvae found in natural pools [43]. Larvae were likely food limited to some extent; food limitation is common under such densities [35]. Prior to the beginning of the experiment, in order to maintain visible identity of clipped versus non-clipped individuals, all the larvae were anesthetized using MS-222 (Tricaine methanesulfonate) and marked according to treatment with Visible Implant Elastomer (VIE) color tags injected subcutaneously (Northwest Marine Technology, Inc.). From each individual in the clipped tail treatment, we removed ~30% (horizontal maximum length: 4–5 mm) of the tail tissue from the posterior end, applying a vertical incision with a sterilized scalpel. We maintained a constant density by replenishing missing larvae with new larvae marked with different color tags. Missing larvae with clipped tails were replaced with clipped tail larvae and missing larvae with intact tails were replaced with larvae with intact tails. These supplemental individuals were not included in the final analysis. We used the term 'missing' because determining the cause of larval death was beyond the scope of this study.

We monitored larval survival at 10, 30, 50, and 60 day from the start of the experiment by emptying the tub water through a D-net. Additionally, we measured time to, size at, and survival to metamorphosis. From the time the first larvae metamorphosed (7 April 2014) until the last larva metamorphosed (7 May 2014), we measured metamorphs length using a digital caliper (± 0.01mm) and wet mass using an electronic balance (± 0.001g).

We used SPSS version 21.0 (SPSS IBM, New York, U.S.A) to perform ANOVA of larval size prior to treatment application, and on larval metamorphic variables and a Tukey HSD post hoc test to identify the sites of origin that differed in size and weight at metamorphosis. We used repeated measures nested ANOVA on larval survival.

## Results

### Laboratory Experiment

In the laboratory study, after clipping an average 28.19% (SE = 3.8) of the tail area, an average of 10.6% (SE = 0.88) had regenerated after 29.3 (SE = 2.5) days. There was no significant difference in daily regeneration rate between the larvae originating from Tel-Dan (M = 0.015, SE = 0.002) and the larvae originating from Manof (M = 0.02, SE = 0.002) (t_14_ = 1.65; p = 0.122). However, daily tail regeneration rate as a function of larval age varied non-linearly showing a hump-shape response, with a peak regeneration rate at~30 days (Fig 2). A polynomial 2^nd^ degree curve explained48.3% of the variation in the data (F_2, 13_ = 6.07, p = 0.014), (Fig 2).

### Outdoor Mesocosm Experiment

In the outdoor mesocosm experiment, prior to tail clipping, we found no significant differences between the larvae in the two treatment groups for wet mass (F_1, 14_ = 0.99; p = 0.336), snout-tail length (F_1, 14_ = 0.79; p = 0.391), or snout-vent length (F_1, 14_ = 0.47; p = 0.500). We found a significant effect of site of origin on larval wet mass (F_4, 14_ = 57.52; p<0.001), snout tail length (F_4, 14_ = 50.46; p<0.001), and snout-vent length (F_4, 14_ = 56.24; p<0.001). A Tukey HSD post hoc test indicated that larvae originating from the temporary sites Manof and Secher were significantly lighter and smaller compared to the larvae originating from the permanent breeding sites: Ein Alon, Ein el Balad, and Kaukab Springs.

**Fig 3:**
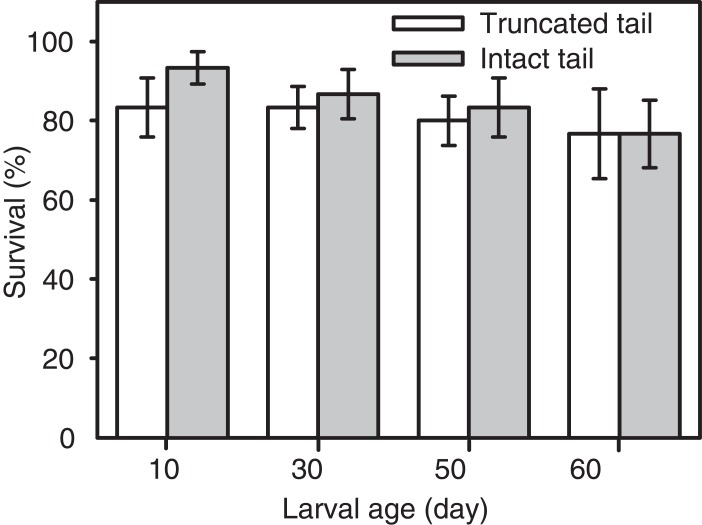
Tail clipping effects on survival of *Salamandra infraimmaculata* larvae. Mean (±SE) larval survival per tub 10, 30, 50, and 60 days from the start of the experiment.

A repeated measures nested ANOVA indicated no effect of tail clipping (F_1, 24_ = 0.75; p = 0.396), site (F_8, 24_ = 1.66; p = 0.161), time (F_3, 24_ = 1.07; p = 0.382), or time by treatment interaction (F_3, 24_ = 0.19; p = 0.903) on larval survival (Fig 3).

**Fig 4:**
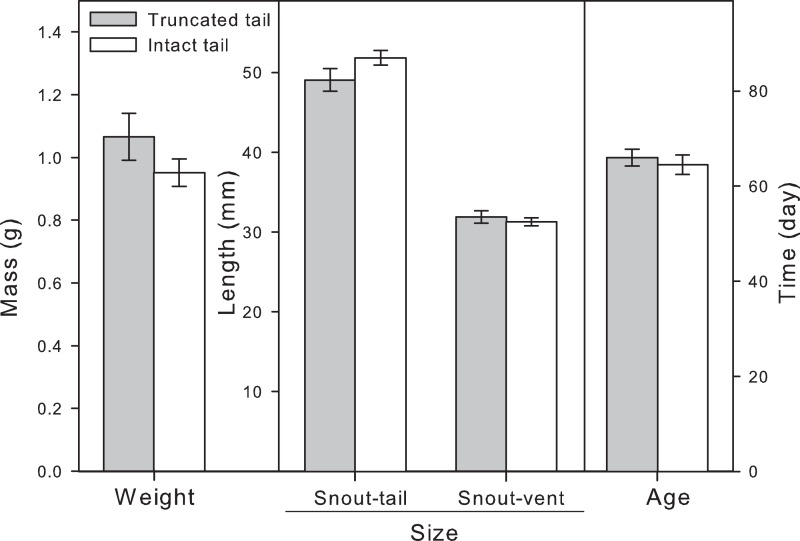
Tail clipping effects on larval *Salamandra infraimmaculata* mean (±SE) mass, snout-tail length, snout-vent length, and time to metamorphosis (Truncated tail [white histogram bars]: N = 20; Intact tail [gray histogram bars]: N = 18).

An ANOVA on the larval metamorphic variables indicated no significant difference between clipped and intact tail larvae. In fact, mass at metamorphosis (p = 0.071) and snout-vent length at metamorphosis (p = 0.195) tended to be larger for the clipped individuals. Snout-tail length at metamorphosis (p = 0.203) and time to metamorphosis (p = 0.709) were also not statistically significant (Table 1; Fig 4). We found a significant effect of site of origin on mass at metamorphosis (p = 0.009), snout-tail length at metamorphosis (p = 0.005), snout-vent length at metamorphosis (p = 0.002), but not on time to metamorphosis (p = 0.271). Site x treatment interaction were non-significant (p>0.05) for all metamorphic variables (Table 1). A Tukey HSD post hoc test indicated that larvae originating from the temporary pool Manof were significantly lighter and smaller when metamorphosed compared to the larvae originating from the permanent breeding sites, Ein Alon (Weight: p = 0.014; STL: p = 0.013; SVL: p = 0.006), and Ein el Balad (Weight: p = 0.017; STL: p = 0.007; SVL: p = 0.004).

**Table 1:** ANOVA assessing the effect of treatment, site, and the interaction between site x treatment on larval weight, snout-tail length (STL), snout-vent length (SVL), and time to metamorphosis.

	Mass		STL		SVL		Time	
**Treatment**	**F1, 28**	**p**	**F1, 28**	**p**	**F1, 28**	**p**	**F1, 28**	**P**
	3.53	0.071	1.7	0.203	1.76	0.195	0.14	0.709
**Site**	**F4, 28**	**p**	**F4, 28**	**p**	**F4, 28**	**p**	**F4, 28**	**P**
	4.16	0.009	4.66	0.005	5.37	0.002	1.37	0.271
**Treat x Site**	**F4, 28**	**p**	**F4, 28**	**p**	**F4, 28**	**p**	**F4, 28**	**P**
	1.41	0.256	0.73	0.579	1.08	0.384	1.2	0.334

## Discussion

Our study contributes to the understanding of costs associated with natural tail damage. It also has implications for use of tail clipping in population studies and genetic studies in urodeles, particularly for endangered species. Unraveling costs associated with the reduction in larval performance due to damaged appendages and costs related to reallocation of metabolic resources associated with regeneration can prove challenging for both ecologists and developmental biologists. The results of our laboratory experiment suggest that, free from predation and competition (individually reared) and constraints associated with limited food resources (*ad libitum* feeding), rates of tail regeneration in *S*. *inframmaculata* larvae still vary throughout their pre-metamorphic developmental phase. Typical growth curves of *Salamandra* larvae do not show that hump-shape curve [44]. The peak in tail regeneration rate at the age of ~30 days (Fig 2) suggests tradeoffs in resource allocation between regeneration and somatic growth during early and advanced development. Tail regeneration in juvenile leopard geckos (*Eublepharis macularius*) where the tail serves as energy storage shows priority over growth (increase in body mass and SVL) following autotomy [45]. Alternatively, variation in regeneration rate may be related to regulation of thyroid hormone. For example, in amputation in zebrafish caused an enhanced expression of type 3 deiodinase (D3) that can decrease thyroid hormone (T3) concentrations in progenitor cells to allow their proliferation [46]. Ontogenetic changes in tail function may explain variation in fitness associated with costs of tail damage and tail regeneration at different life stages. For many terrestrial urodeles, a pronounced change in the tail function occurs post-metamorphosis from a thrust-generating appendage to an energy storage reserve [29]. Regenerative outgrowth in a post-metamorphic urodele was found to depend on the longitudinal position of amputation (proximal versus distal) [47] and on tail width at the point of amputation [48]. Age-specific variation in the costs of tail loss has been suggested for juvenile (low costs) and adult (high costs) lizards in the genus *Eumeces* [49].

Age can affect appendage regeneration at the tissue, cellular, and genomic level [50]. Nevertheless, the cellular and molecular bases for the variation in regenerative capacities across an individuals' lifetime are currently unclear [51]. The ability to repeatedly regenerate fins in zebrafish (*Danio rerio*) declines with age and is correlated with reduced activity and expression of telomerase in aged fish [52]. In tadpoles of the African clawed frog (*Xenopus laevis*), a refractory period appears at an early developmental stage that is characterized by a very low regenerative ability [53]. In amphibians, the metabolic costs of metamorphosis may constrain physiological processes associated with appendage regeneration shortly before the onset of metamorphosis. Nevertheless these costs may be lower in urodeles for which tail structure is not completely reabsorbed during metamorphosis.

Evidence regarding costs associated with tail injury in aquatic larval stages in urodeles are limited and inconclusive. In the cannibalistic mole salamander (*Ambystoma talpoideum*), initial larval density affected the frequency of damaged tails but not the extent of the damage to the tail [28]. In the four toed salamander (*Hemidactylium scutatum*), tail clipping had no effect on larval time to and size at metamorphosis of individually reared larvae in the laboratory, but reduced tail resorption and increased tail growth rate post metamorphosis [29]. In a recent study on California tiger salamander (*Ambystoma californiense*) clipping of up to 50% of tail area had no effect on larval survival, mass, or snout-vent length [27]. The results of our outdoor artificial pool experiment suggest there was no effect of tail clipping on larval age-related survival (Fig 3). Survival declined with time, probably due to conspecific predation but mortality rates were not higher shortly after tail clipping. Though not statistically significant, there was a trend for lower survival of truncated tail larvae compared to intact tail larvae in early developmental stages. The difference in survival rates between the two treatment groups decrease with time from the onset of the experiment when tails were clipped. As the interval between tail clipping and survival sampling increased, truncated tails had ample time to regenerate and resume full or partial functionality.

The results of the outdoor artificial pool experiment also suggest there were no developmental costs to tail clipping as apparent from the lack of difference in time to metamorphosis of clipped versus intact tail larvae. We also failed to find support for growth-related costs, as apparent from the similar size (length and mass) at metamorphosis of larvae in the two treatment groups. The lack of an apparent effect of tail clipping may arise from our experimental setup. Larvae used for the mesocosm experiment were deposited in the laboratory by females collected from two ephemeral breeding sites and three permanent breeding sites. The effect of 'site' on larval weight and size at metamorphosis, i.e. smaller size of the Manof temporary site compared to two permanent sites is consistent with the notion of local adaptation. We attempted to simulate the ecologically relevant conditions for larval growth and development by the use of mesocosms and controlled density and feeding regime. Nevertheless, the intensity and nature of competition and predation interactions in natural larval breeding habitats may vary throughout the larval aquatic phase, for example, due to priority effects of earlier cohorts, and exposure to intraguild predators [35,54].

*S*. *infraimmaculata* larvae may possess a degree of phenotypic plasticity that allow them to compensate for the costs associated with tail injury even before the complete regeneration of the damaged tail and when tail regeneration rate is suboptimal, i.e. at early and late developmental stages. The results of our study suggest that tail clipping of up to 30% of the tail area represent a negligible fitness burden for the larvae. Tail clipping then can be an imperative tool promoting amphibian research in general and particularly the study of conservation-sensitive species and populations where sacrificing larvae is not permitted or an unwarranted option.
