# Correction: Effects of Tail Clipping on Larval Performance and Tail Regeneration Rates in the Near Eastern Fire Salamander, *Salamandra infraimmaculata*

**DOI:** 10.1371/journal.pone.0135839

**Published:** 2015-08-19

**Authors:** Ori Segev, Antonina Polevikove, Lior Blank, Daniel Goedbloed, Eliane Küpfer, Anna Gershberg, Avi Koplovich, Leon Blaustein

Fig 2 incorrectly appears as a duplicate of Fig 3. The authors have provided a corrected version here.

**Fig 2:**
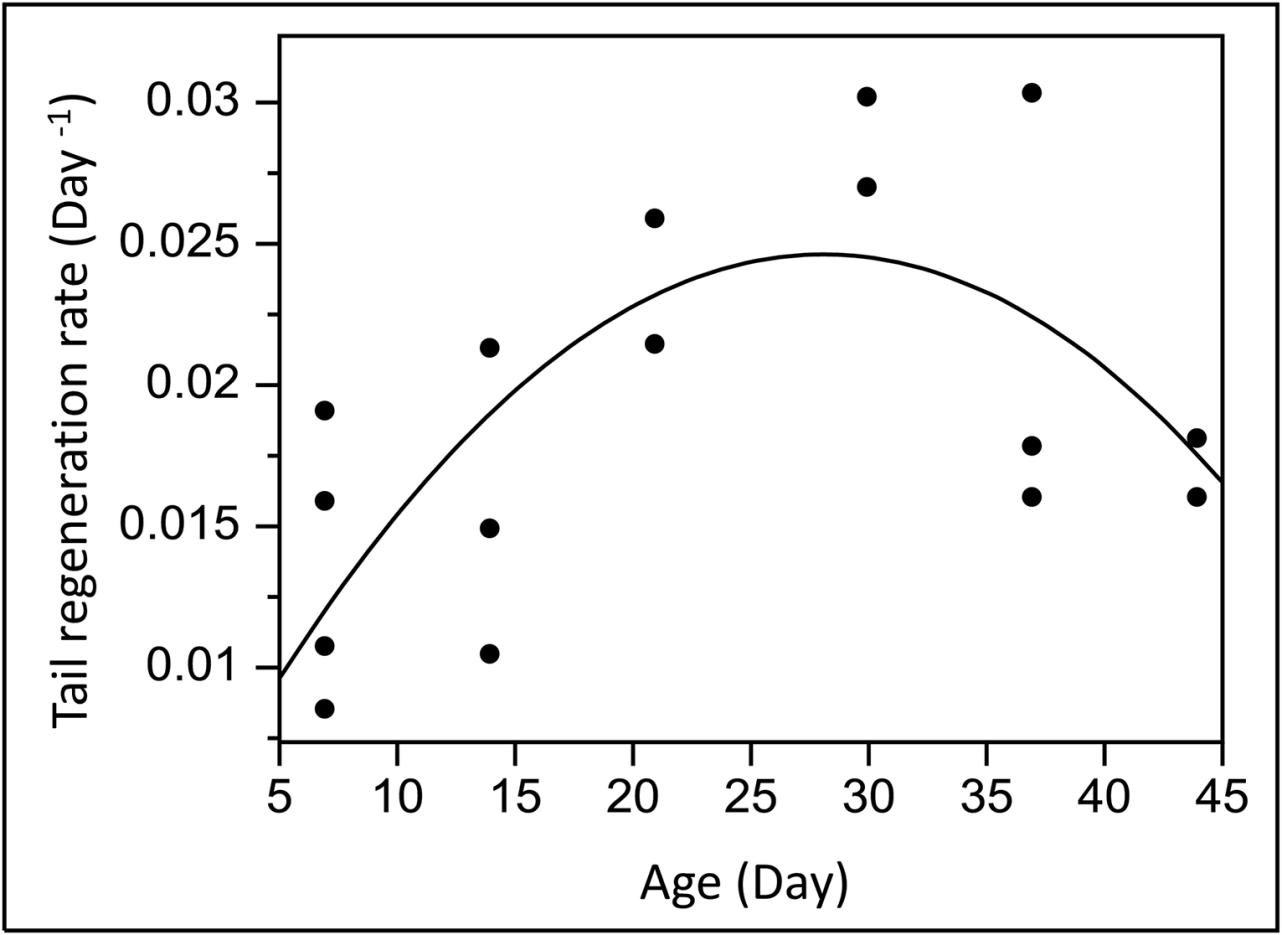
Tail regeneration rate per day of *Salamandra infraimmaculata*larvae versus larval age (day) at the time of tail clipping (N = 16). A polynomial 2^nd^ degree curve explained 48.3% of the variation in the data (F_2, 13_ = 6.07, p = 0.014).
